# Chitosan Hydrogel Doped with PEG-PLA Nanoparticles for the Local Delivery of miRNA-146a to Treat Allergic Rhinitis

**DOI:** 10.3390/pharmaceutics12100907

**Published:** 2020-09-23

**Authors:** Yu Su, Bixi Sun, Xiaoshu Gao, Shuwen Liu, Rubin Hao, Bing Han

**Affiliations:** Department of Biopharmacy, School of Pharmaceutical Sciences, Jilin University, Changchun 130021, China; suyu18@mails.jlu.edu.cn (Y.S.); sunbq19@mails.jlu.edu.cn (B.S.); xs_gao@126.com (X.G.); liushuwenjlu@126.com (S.L.); rubinhao@163.com (R.H.)

**Keywords:** nanoparticles, binary formulation, allergic rhinitis, miR-146a

## Abstract

To prepare a binary formulation delivering miRNA-146 and evaluate a nucleic acid nasal delivery system by investigating its pharmacodynamic effects in allergic rhinitis. The gel/NPs/miR-146a thermosensitive in situ chitosan hydrogel carrying a nucleic acid was prepared and evaluated for its characteristics, including temperature sensitivity, gel strength, mucosal adhesion and drug release profile. After nasal administration of the formulation to ovalbumin-sensitized rats, the treatment of allergic rhinitis was verified by assessing nasal symptoms, hematology, hematoxylin-eosin (HE) staining and immunohistochemistry. Western Blot(WB) was used to analyze nasal inflammatory factors as well as miRNA-146-related factors, and the miR146 expression level was measured by PCR. Subsequently, the effects of the gel/NPs/miR-146a binary formulation were evaluated for the nasal delivery of nucleic acids in rhinitis therapy. The prepared binary formulation quickly formed a gel in the nasal cavity at a temperature of 34 °C with good mucosal adhesion, which delivered nucleic acids into the nasal mucosa stably and continuously. Gel/NPs/miR-146a was able to sustain the delivery of miRNA into the mucosa after nasal administration. When compared with the monolithic formulations, the gel/NPs/miR-146a binary formulation performed better regarding its nucleic acid delivery ability and pharmacodynamic effects. The gel/NPs/miR-146a binary preparation has a suitable nasal mucosal drug delivery ability and has a positive pharmacodynamic effect for the treatment of ovalbumin-induced rhinitis in rats. It can serve as a potential nucleic acid delivery platform for the treatment of allergic rhinitis.

## 1. Introduction

Allergic rhinitis (AR) is a type I allergic inflammatory disease that is of the nasal mucosa induced by Immunoglobulin E (IgE) under the chemotaxis of a variety of inflammatory cytokines after a specific individual comes in contact with antigens. Studies have shown that after exposure to allergens, there are changes in the body, such as T cell subpopulation imbalance and abnormal inflammatory cytokine levels, resulting in an imbalance of cell/immune responses between Th1 and Th2. Th2 cytokines stimulate B-lymphocytes to produce IgE [1]. After IgE binds to the antigen, it activates basophils and mast cells, releases histamine from the cytoplasm, increases vascular permeability, contracts bronchial smooth muscles and activates sensory nerves, resulting in sneezing and nasal itching symptoms [2]. The prevalence of AR is cited as 10% to 30% in adults and up to 40% in children [3]. AR undoubtedly brings many inconveniences to the daily lives of patients. Commonly used antiallergic drugs cannot eradicate the disease. Although allergen immunotherapy is effective, it has the disadvantage of a limited number of allergens [4]. Regulation of AR inflammation and the immune system based on miRNA regulation is a potential intervention method [5].

MicroRNA (miRNA) is a type of noncoding single-stranded RNA that can target RNA in animals and plants to prevent protein translation or degrade mRNA and participates in posttranscriptional gene expression regulation. miRNAs play a vital role as gene regulators in almost every aspect of controlling cellular functions. Moreover, it has been shown that they actively participate in immune function and allergic inflammation [6,7]. Previous studies have shown that miR-146a is involved in suppressing inflammation, regulating the immune system and suppressing allergic inflammation [8,9]. Existing studies have indicated that miR-146a has an important therapeutic effect on the induction of immune tolerance in AR and is considered a promising biomarker in the pathogenesis and treatment of AR [10,11]. However, naked miRNA has difficulty penetrating the extracellular barrier and is easily degraded in vivo. In addition, it often has a short retention time because of removal by the nasal cilia, reducing the contact time of the drug with nasal epithelial cells [12]. Therefore, it is necessary to select an appropriate delivery system to promote cellular uptake and protect miRNAs from degradation, thereby improving bioavailability.

Chitosan is biodegradable, safe, nontoxic and mucoadhesive; accordingly, it can be used as a hydrogel matrix for local intranasal administration in order to prolong the retention time of drugs in the nose [13,14,15]. Considering the enrichment of multiple enzymes in the nasal cavity, delivery of nucleic acids in the form of a chitosan hydrogel alone may be susceptible to the risk of instability in the nasal cavity [16]. In addition, the drug carrier for local intranasal treatment should have suitable mucopenetrating ability to achieve sustained and stable drug delivery to the nasal mucosa. polyethylene glycol (PEG)-poly(lactic acid) (PLA) nanoparticles have the proper ability for nucleic acid delivery and maintain nucleic acid stability in vivo as well [17]. The presence of PEG chains on its surface can enhance the permeability of mucus, allowing the drug to pass through the mucus layer and then transporting the drug to the mucosa [18]. Various nanotechnology-based systems, such as liposomes, polymeric nanoparticles, dendrimers, cyclodextrin complexes, gold nanoparticles and magnetic nanoparticles, are being explored in the pursuit to improve aqueous solubility and drug delivery to the pathological site [19,20].

**Scheme 1:**
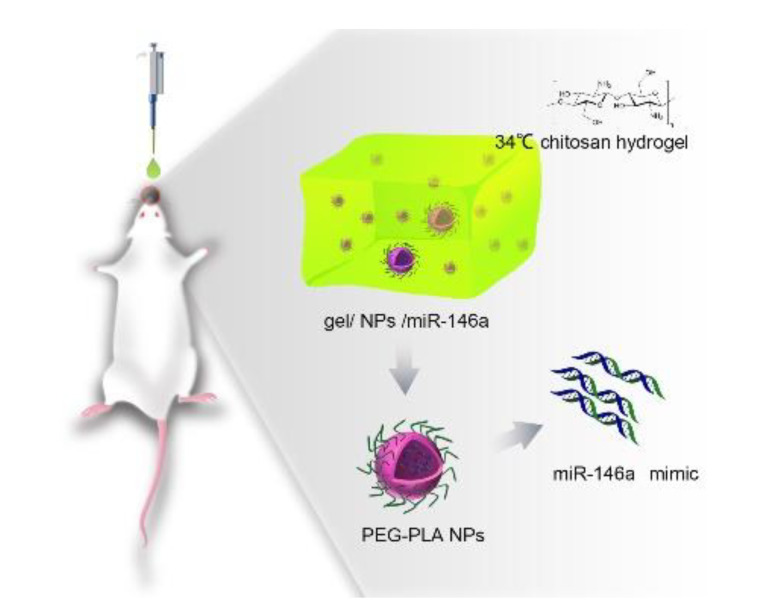
The miRNA-NPs/CS temperature-sensitive hydrogel steadily delivers miRNA-146 into the nasal mucosa.

To remove the limitation of applying miRNA in the nasal mucosa, we sought to design a safe and stable formulation to deliver miR-146afor the local treatment of allergic rhinitis. According to the unique pH (5.5–7.8) and temperature (32–35 °C) of the nasal cavity [21,22,23], a thermosensitive in situ hydrogel was designed that is liquid at room temperature for easy preparation and administration with a controllable dosage (Scheme 1). After reaching the lesion area, it should be transformed into gel to increase retention time. Our research has proven that the temperature-sensitive PEG-PLA NPs/hydrogel binary formulation has appropriate nasal mucosal adhesion properties. After nasal administration, the miRNA-NPs/CP temperature-sensitive hydrogel steadily delivers miRNA-146 into the nasal mucosa, which significantly inhibits inflammation in the rat allergic rhinitis model, and causes pathological changes to the nasal mucosa, leading to an excellent therapeutic effect. The rationally designed miRNA-NPs/CP thermosensitive hydrogel binary formulation provides the potential for realizing nucleic acid drug therapy for nasal diseases.

## 2. Materials and Methods

### 2.1. Materials

Ovalbumin (OVA) was obtained from Sigma-Aldrich (Saint Louis, MO, USA). The antigen adjuvant suspension was composed of 1 mg of ovalbumin and 50 mL of aluminum hydroxide gel. PEG5000-PLA75/25 was purchased from Daigang Biotechnology Co., Ltd. (Shandong, China). Chitosan (viscosity of 100–200 mpa.s and a degree of deacetylation of 95%) and β-GP were purchased from Macklin Biochemical Technology Co., Ltd. (Shanghai, China). Cy3-miRNA-146a was provided by Gene Pharma (Shanghai, China). All reagents, unless otherwise specified, were purchased from Xinjinji Biotechnology Co., Ltd. (Jilin, China). The sources of the instruments used are indicated in the corresponding position.

### 2.2. Animals

The Sprague Dawley rats used (weight 220–250 g) in this study were purchased from Changsheng Biotechnology Co., Ltd. (Changchun, China). The rats were given free access to OVA-free water and food before the experiment. The experimental procedure was approved by the Ethics Committee of Jilin University. All animal studies were conducted in strict accordance with the approval of the Animal Ethics Committee of the Pharmacy of Jilin University (code: 20180027).

### 2.3. Preparation and Characterization of miR146a/PEG-PLA NPs

Nanoparticles were prepared by the double emulsion method [24,25]. The operation process was as follows. First, miR-146a was added into enzyme-free water containing spermidine (N/P ratio 10:1) with stirring to form a spermidine-miR146a complex. PEG-PLA (50 mg) and the spermidine/miR-146a complex were dissolved in dichloromethane (2 mL). The obtained organic solution was poured into 25 mL of PVA aqueous solution (2%, *w*/*v*) and the mixture was sonicated for 60 s in an ice water bath using a probe for emulsification. The nanoparticles were centrifuged at 8000 rpm for 20 min and collected. The particle size and zeta potential of the nanoparticles were measured and analyzed by a dynamic light scattering particle size analyzer (Nano-ZS90, Malvern, UK). The shape and surface morphology of the nanoparticles were observed by transmission electron microscopy (TEM JEM-2200FS, JEOL, Tokyo, Japan).

Using a microplate reader (λex/em = 550/570 nm), (Infinite F200Pro, TECAN, Männedorf, Switzerland), the miRNA in the supernatant was quantified according to the calibration curve of miR-146a, and all experiments were repeated three times. The loading efficiency of miRNA was calculated as follows [26]:

(1) $$Loading\;efficiency\;of\;miRNA=\frac{\left(initial\;amount\;of\;miR-amount\;of\;miR\;in\;supernatant\right)}{initial\;amount\;of\;miR}\times 100\%$$

### 2.4. Preparation of a Thermosensitive In Situ Gel

Sodium glycerophosphate/chitosan was used to prepare composite hydrogels. The preparation method was described in previous reports [27]. Briefly, deacetylated chitosan was dissolved in 1% acetic acid at various concentrations of 1.5%, 3% and 4.5% (*w*/*v*). To prepare the hydrogel, 2 mL of chitosan solution was continuously stirred at 4 °C as the prepared nanoparticles (or same equivalent of naked miR-146a 0.07 mg) were slowly added. Then, a β-GP (12% or 16% *w*/*v*) solution was added dropwise. The mixed solution was stirred at room temperature for half an hour.

### 2.5. Determination of Gelation Time, Gel Strength and Mucosal Adhesion

The gelation time was measured by the rod stop method with some modifications [28]. A 20 mL beaker was placed on a heated magnetic stirrer (Li chen ke yi, Shanghai, China) and 5 mL of the preparation was added. The liquid was constantly stirred (30 rpm) at 34 °C. When the magnetic rod stopped moving due to gelation, the time was recorded. Three independent experiments were performed in parallel, and the results are the average of the measurements.

Gel strength was determined as the time required for the weight to penetrate 5 cm deep into the gel. Each sample (50 g) was transferred to a 100 mL graduated cylinder and gelled in a constant temperature water bath at 34 °C. Then, a 35 g weight was placed on the gel solution and allowed to penetrate 5 cm in the gel. Time taken by weight to sink 5 cm was measured [29]. 

Mucosal adhesion gel strength was based on the two-arm balance method [28]. At 34 °C, a piece of porcine nose mucosa was stacked on the opening of a glass vial filled with phosphate buffer saline (PBS) (pH 7.4). The glass vial was glued tightly to the center of a beaker containing phosphate buffer (pH 7.4, 34 °C). The fixed-dose preparation was gelled and fixed on the underside of the rubber stopper in advance. The stress required to separate the gel from the mucosal surface per unit area was determined to be the mucosal adhesion strength (g/cm^2^).

The method for determination of the adhesion time between the gel preparation and mucosa was based on that by Khan et al. [30] and slightly modified. At 34 °C, a pregelatinized sample (2 g) with 0.1% methylene blue was added to a sample, and the sample was adhered to porcine nose mucosa. The sample was placed in a constant temperature environment of 34 °C at an angle of 40° and rinsed with the nasal fluid of a simulated patient at pH 7.4 and a rate of 5 mL/min. Based on the color change, the time required to thoroughly wash out the formulation was determined. The results of all the above experiments are shown as the average of 4 independent measurements.

### 2.6. In Vitro miRNA Release from PEG-PLA Nanoparticles

This test was carried out by the dialysis bag method. PEG-PLA nanoparticles containing Cy3-labeled miRNA were placed in 1 mL of PBS and released into a water bath at 34 °C and 100 rpm. The sample of the supernatant of each formulation was removed at 0 h, 4 h, 8 h, 16 h, 24 h, 32 h, 40 h and 48 h, and replaced with fresh buffer.

The fluorescence intensity of each sample was measured at 550/570 nm with a microplate reader, the amount of miRNA released was determined and PBS solution was used as a blank control.

### 2.7. Ex Vivo Nasal Mucosal Penetration and Retention of miRNA

The ex vivo nasal mucosal penetration experiment used the Franz vertical diffusion cell method. In short, we first prepared the ex vivo nasal mucosa (code: 20180043). The nasal cavity was removed from the nose of a freshly slaughtered pig, and tweezers were used to carefully peel off the mucosa covering the turbinate bone and nasal septum, followed by rinsing with normal saline. The treated nasal mucosa was fixed on the diffusion cell (the penetration area was 3.14 cm^2^). Five grams of the sample to be tested were paced in the supply tank and evenly covered the surface of the nasal mucosa. Then, the cell was sealed with plastic wrap, fixed with 19 mL of normal saline (34 ± 0.2 °C) in the receiving tank and allowed to come into close contact with the nasal mucosa. The diffusion device was placed in a constant temperature bath at 34 ± 0.2 °C, stirred with a magnetic stirrer (speed of 300 r/min) and a 1 mL sample was removed at 0 h, 4 h, 8 h,16 h, 24 h, 32 h, 40 h and 48 h, and the permeated miR-146a of the different preparations was determined. The permeation of the Cy3-labeled miRNA into the mucosa was plotted with time T as the abscissa and cumulative permeation per unit area Q (μg/cm^2^) as the ordinate. After 48 h, the amount of miRNA retained in the nasal mucosa was determined as follows:

(2) $$\begin{array}{l} Amount\;miRNA\;retained\\ \;\;\;\;\;\;\;=Total\;miRNA\;content-miRNA\;content\;in\;diffusion\;pool\\ \;\;\;\;\;\;\;-miRNA\;content\;in\;receiving\;pool \end{array}$$

### 2.8. Preparation of AR Model Rats and Intranasal miR-146a Administration

Thirty-six rats were randomly divided into 6 groups: the normal group, AR model group, naked miR-146a group, AR + gel/miR-146a group, AR + NPs/miR146a group and AR + gel/NPs/miR-146a group (miR-146a: 1.4 × 10^−3^ mg/rat) [10]. According to previous reports, the AR model was induced by ovalbumin sensitization. With the prepared allergen suspension containing 0.3 mg OVA + gel 30 mg AL(OH)_3_, 1 mL per mouse was injected intraperitoneally once every other day for a total of 7 administrations as the basic sensitization. From day 15 to day 21, for 7 consecutive days, 50 μL of OVA (25 mg/mL) was applied to each nostril via a micropipette once a day. The rats were administered this treatment for 7 consecutive days after the 22nd day. The rats were fixed during the nasal drip and returned to the cage after 10 min of continuous fixation. The model group was given only sterile saline each time, and approximately 20 μL of miR-146a, NPs/miR-146a, gel/miR-146a and gel/NPs/miR-146a containing 7 × 10^−4^ of miR-146a was introduced in one of the nostrils of each rat with a micropipette. Rats in the normal group were injected intraperitoneally with the same amount of normal saline and nasal drops. On the 30th day, the 6 groups of rats were sacrificed by anesthesia, and the mucosal tissues were collected for further examination.

### 2.9. Evaluation of Rhinitis Symptoms

According to reference [31], all rats were evaluated for their nasal symptoms as described by Wen et al. On the 30th day, each rat was placed in a separate transparent cage, and after half an hour of adaptation, sneezing, nasal scratching and secretions from the nose were monitored for 10 min. No nose scratching or sneezing received a score of 0, the behaviors 1–3 times/min received a score of 1, the behaviors 4–6 times/min received a score of 2 and the behaviors exceeding 6 times/min received a score of 3. No nasal secretions were scored as 0 points, a single nostril secretion was scored as 1 point, two nostril secretions were scored as 2 points, and an overflow volume was scored as 3 points. From these scores, the total score of nasal symptoms for each rat was calculated and compared.

### 2.10. Enzyme-Linked Immunosorbent Assay (ELISA)

The levels of the serums IL-4, IL-13, TNF-α, IFN-γ and IgE and histamine were determined by an enzyme-linked immunosorbent assay. Each sample was measured in triplicate, and the average value was recorded. The levels of IL-4, IL-13, TNF-α, IFN-γ, IgE and histamine were determined according to the ELISA kit instructions. The absorbance was measured at 450 nm with a microplate reader (Infinite F200Pro, TECAN, Männedorf, Switzerland). Each measurement was repeated in triplicate, and the average value was recorded. Ig-E, IL-4, TNF-α and INF-γ levels were expressed as pg/mL and IL-13 levels as ng/mL.

### 2.11. Apoptotic Cell Detection in Nasal Mucosa

The TUNEL method was used to detect cell apoptosis in the nasal mucosa. According to the kit instructions, the rat nasal mucosa was fixed with 10% neutral formaldehyde, embedded in paraffin, dewaxed with conventional xylene and added to a gradient of ethanol in water. Cell apoptosis was observed under a 400× light microscope and 6 different fields of view were randomly selected from each slice to calculate the cell apoptosis rate of each group of nasal mucosa. The nuclei of apoptotic cells were stained brown, normal cells were blue and apoptotic cells were brown.

### 2.12. qRT-PCR

Total RNA was extracted from the nasal mucosa using TRIzol reagent. cDNA was synthesized using a Prime ScriptTM reverse transcription kit (Invitrogen, NY, USA). The expression of mRNA was quantified by real-time RT-qPCR. The primer sequences used were as follows (5′ to 3′): miR-146a: 5′-CTGCCGCTGAGAACTGAATT-3′ (forward), 5′-CAGAAGCAGGGTCCGAGGTA-3′ (reverse); U6: 5′-CTCGCTTCGGCAGCACA-3′ (forward), 5′-AACGCTTCACGAATTTGCGT-3′ (reverse). The data were analyzed using the 2-ΔΔCT method.

### 2.13. Western Blot Assay

The classical proteins TLR4 and NF-κB, which are associated with rhinitis, were detected to indirectly investigate the nucleic acid delivery effects of each group of nanoparticles. Specifically, nasal mucosa tissue cells were collected in a RIPA Lysis Buffer (RIPA) buffer containing protease inhibitors and protein samples were collected for SDS-PAGE. After blocking with 5% skim milk for 1 h at room temperature, the primary antibody was added and followed by incubation overnight at 4 °C. After incubating the secondary antibody bound to Horseradish Peroxidase (HRP) for 2 h, the membrane was washed with PBS containing 0.1% Tween-20. ImageJ software (National Institutes of Health, Bethesda, MD, USA) was used to analyze the gray value.

### 2.14. HE Staining

After the rats were sacrificed, the maxillary skin was stripped, freed from the skull and cut along the midline of the nose to expose the nasal septum and bilateral nasal cavities. After administration for 1 week, the bilateral nasal mucosa tissues were taken immediately and treated with PBS 3 times, fixed with 4% paraformaldehyde, embedded in conventional paraffin and sectioned at a thickness of approximately 5 μm. After dewaxing with xylene and washing with a gradient of ethanol to water, the sections were stained with hematoxylin-eosin (HE). Then, the sections were routinely dehydrated, made transparent and resin-mounted, and the histopathological changes in the nasal mucosa were observed under a microscope (CKX41, Olympus, Tokyo, Japan).

### 2.15. Immunohistochemistry

The classical protein factors associated with rhinitis, TLR4 and NF-κB, were further studied. Immunohistochemical analysis of the nasal mucosa was performed to assess TLR4 and NF-κB expression in the nasal mucosa after initiation of rhinitis in the normal group, model group and groups with different formulations delivering miR-146a. The miR-146a delivery effects of each formulation were evaluated indirectly.

### 2.16. Statistical Analysis

Analysis of the data was performed using GraphPad Prism software (GraphPad Software, San Diego, CA, USA). All data are presented as the mean ± standard deviation (SD). One-way ANOVA, followed by Tukey’s test, was used for multiple comparisons. Differences with *p*-values less than 0.05 were considered to be significant.

## 3. Results

### 3.1. Characterization of the PEG-PLA Nanoparticles Carrying miRNA-146a

**Figure 1:**
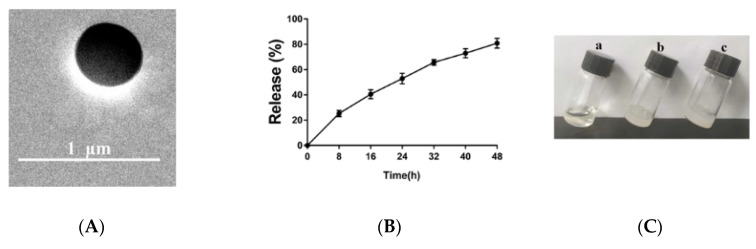
(**A**) Characterization by transmission electron microscopy. (**B**) miR-146a cumulative release curves from the nanoparticles. The fluorescence intensities of each sample collected at each time point were measured, and the fluorescence from the blank control was subtracted. The cumulative release curve was calculated based on the obtained concentration. (**C**) The formulation at room temperature (a, gel/NPs/miR-146a) and at 34 °C (b, gel/miR-146a; c, gel/NPs/miR-146a). The data are expressed as the mean ± SD, *n* = 4.

The average size, zeta potential range and polydispersity index of the nanoparticles were measured by a Malvern particle size analyzer as shown in Figure 1A and Table 1.

### 3.2. The Release Profile of miR-146a In Vitro

Under a simulated nasal environment (34 °C, pH = 7.4), the release profile of miRNA from the nanoparticles was measured. The release curve was constructed by measuring the fluorescence of Cy3-labeled miRNA at different time points. PBS solutions containing only PBS or Cy3-labeled miRNA were used as standard curves. The cumulative release curve of fluorescence intensity was calculated based on the concentration obtained in Figure 1B.

### 3.3. Characterization of the Chitosan Thermosensitive In Situ Hydrogel

**Figure 2:**
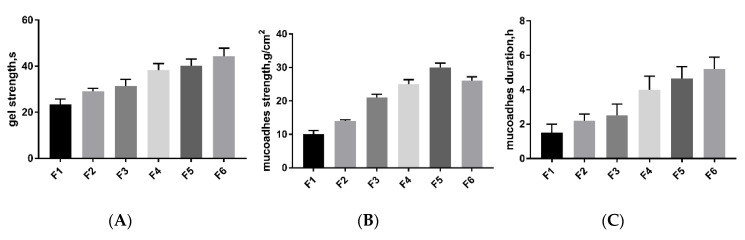
Gel properties. (**A**) Gelation gel strength, (**B**) mucosal adhesion strength and (**C**) mucosal adhesion duration of various thermosensitive mucoadhesive gel preparations (for gel composition, please refer to Table 2). The data represent the mean ± SD (*n* = 4).

We optimized a series of formulations (Table 2) to find formula F5. After mixing 1 mL of a chitosan aqueous solution with 16% *w*/*v* β-GP, the formulation quickly turned into a gel within 3.8 min at a gelling temperature of 34 °C (Figure 1C). Generally, the gel strength of nasal preparations can effectively maintain the integrity of the gel for 20–50 s. The mucosal adhesion strengths of the gel/NPs/miR-146a were 30 ± 1.61 g/cm^2^. It should be noted that there was no significant difference in the mucosal adhesion strength and adhesion duration between the miRNA/chitosan hydrogel and NPs/chitosan hydrogel in the solution and gel state (Figure 2), suggesting that the incorporation of PEG-PLA NPs/miR146a showed little influence on the gel properties (Table 3).

### 3.4. Ex Vivo Nasal Mucosal Penetration and Retentionabsorption Efficiency of miR146a

**Figure 3:**
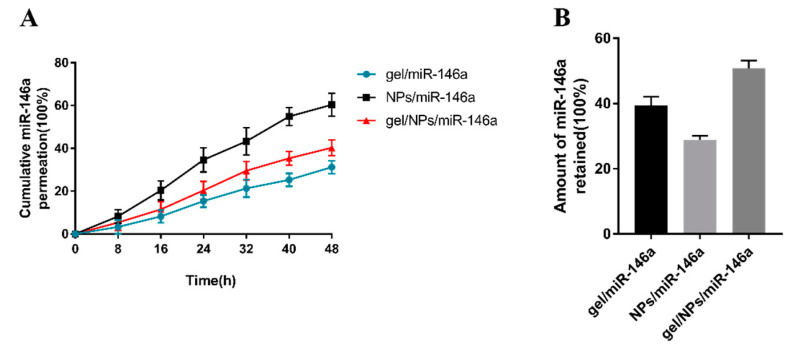
(**A**) Cumulative miR-146a curve of permeation in the nasal mucosa from the NPs/miR-146a, gel/miR-146a, and binary gel/NPs/miR-146a formulations. (**B**) Amount of miR-146a retained in the nasal mucosa after 48 h of application of the different formulations.

The penetration rates of miR-146 from NPs/miR-146a and gel/miR-146a were 61% and 31%, respectively, and the penetration rate of the gel/NPs/miR-146a binary formulation was 40% (Figure 3A). The results showed that the use of delivery vehicles significantly hindered the penetration of miRNA from the mucosa, and the hydrogel formulation had a stronger hindering effect. In order to evaluate the effective dose that actually remained in the inflamed mucosa after the topical application of different formulations in the nose, miRNA was recovered from the mucosa 48 h after administration and delivery. The gel/NPs/miR-146a binary formulation showed the highest mucosal retention rate, with 51% of the initial dose remaining in the mucosa. After 48 h, the mucosal retention of NPs/miR-146a was slightly lower at 29%, and that of the gel/miR-146a was 39% (Figure 3B).

In general, the formulation with the lowest miRNA penetration and the highest interception is more suitable for local nasal mucosa administration; that is to say, the gel/NPs/miR-146a binary formulation might be the optimal choice.

### 3.5. The Effects of miR-146a Delivery on the Behavioral Performance of Rats

**Figure 4:**
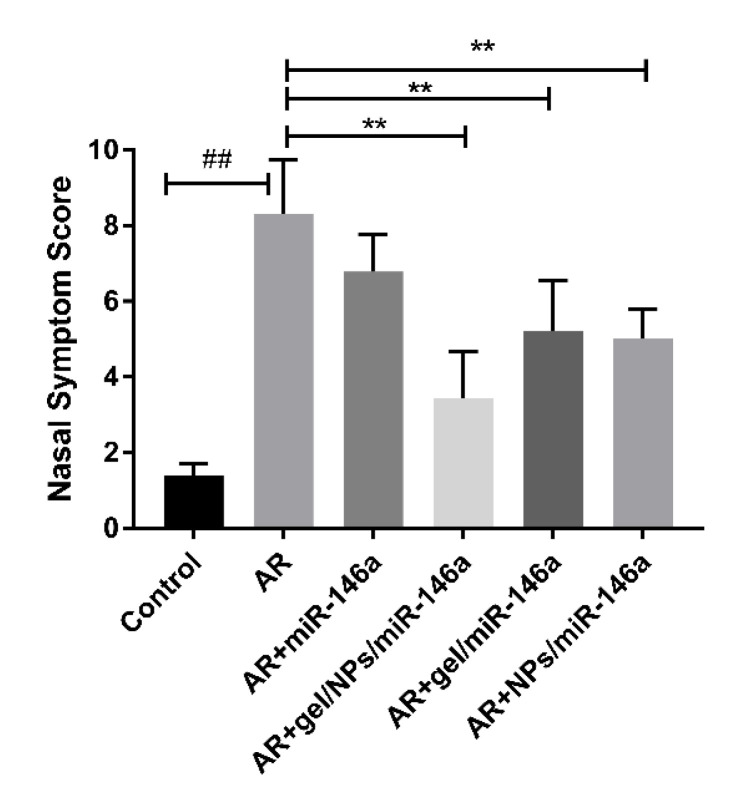
Nasal symptom score results. Each value represents the mean ± SD of the 6 rats in each group. ^##^ *p* < 0.01 compared with the control group; ** *p* < 0.01 compared with the allergic rhinitis (AR) group.

The AR model rats were prepared successfully. Compared with AR model rats, AR + gel/miR-146a, AR + NPs/miR-146a and AR + gel/NPs/miR-146a treated AR rats had a significant reduction in the number of nasal scratches and sneezing. Among them, the AR + gel-NPs-miR-146a group had the best scores, and the treatment effect was the most obvious. While rats in the naked miR-146a group showed a slight improvement in allergic rhinitis symptoms, the effect was not obvious (Figure 4). This may be because naked miR-146a was easily cleared by nasal cilia or degraded by related enzymes in the nasal cavity.

### 3.6. miR-146a Delivery Affects Histamine and Inflammatory Cytokine Levels

**Figure 5:**
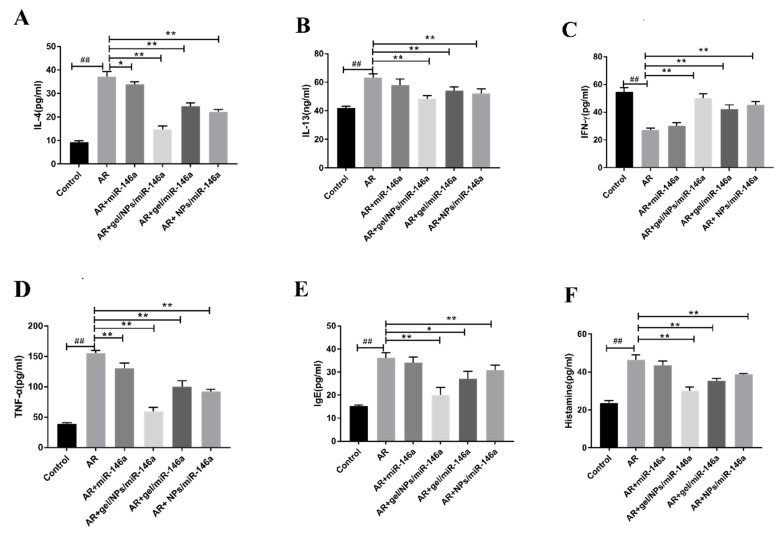
The effects of miR-146a delivered by binary formulations on cytokines in AR rat serum. The contents of (**A**) IL-4, (**B**) IL-13, (**C**) IFN-γ, (**D**) TNF-α, (**E**) IgE and (**F**) histamine in rat serum were determined by ELISA. Data represent the mean ± SD of three independent experiments. ^##^ *p* < 0.01 compared with the control group; * *p* < 0.05, ** *p* < 0.01 compared with the AR group.

We measured the expression levels of IL-4, IL-13, TNF-α, IFN-γ, IgE and histamine, measured by ELISA, which play a key role in AR. Figure 5 shows that compared with the AR model group, the groups after miR-146a administration (AR + naked miR-146a, AR + gel-miR-146a, AR + NPs-miR-146a and AR + gel-NPs-miR-146a) had reduced levels of IL-4, IL-13, TNF-α, IFN-γ, IgE and histamine. Overall, the AR + gel/NPs/miR-146a group exhibited the best pharmacodynamic effect. In summary, these results indicate that AR + gel/NPs/miR-146a showed the best regulation of inflammatory cytokines (Figure 5).

### 3.7. Effect of miR-146a Delivery on Rat Nasal Mucosa Apoptosis

**Figure 6:**
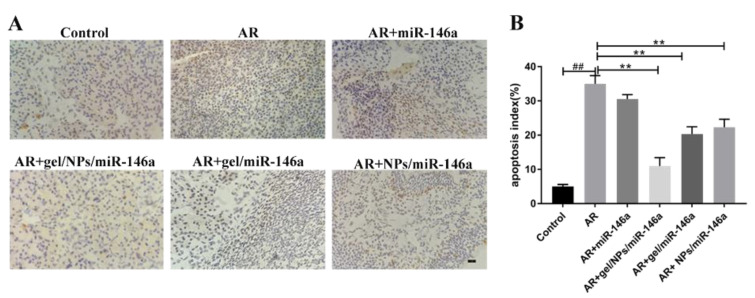
(**A**) Apoptosis of nasal epithelial cells determined by TUNEL staining (400×). (**B**) Quantitative analysis of the apoptosis rate of nasal epithelial cells. Data represent the mean ± SD of three independent experiments. ^##^ *p* < 0.01 compared with the control group; ** *p* < 0.01 compared with the AR group.

TUNEL staining was used to evaluate the effects of nucleic acid delivery by nasal administration of each preparation on nasal mucosal cells (Figure 6). By observing the conditions of the nasal mucosa in each group, it was found that compared with the normal group, the nasal mucosal synovial cell cytoplasm had a large number of brown particles in the model group, showing more nasal mucosal cell apoptosis. Compared with the model group, the number of apoptotic cells in the nasal mucosa of the nucleic acid delivery group and the naked miR-146a group was significantly reduced, among which the number of apoptotic cells in the gel/NPs/miR146a group was the lowest.

### 3.8. miR-146a Expression Levels Were Detected by qRT-PCR

**Figure 7:**
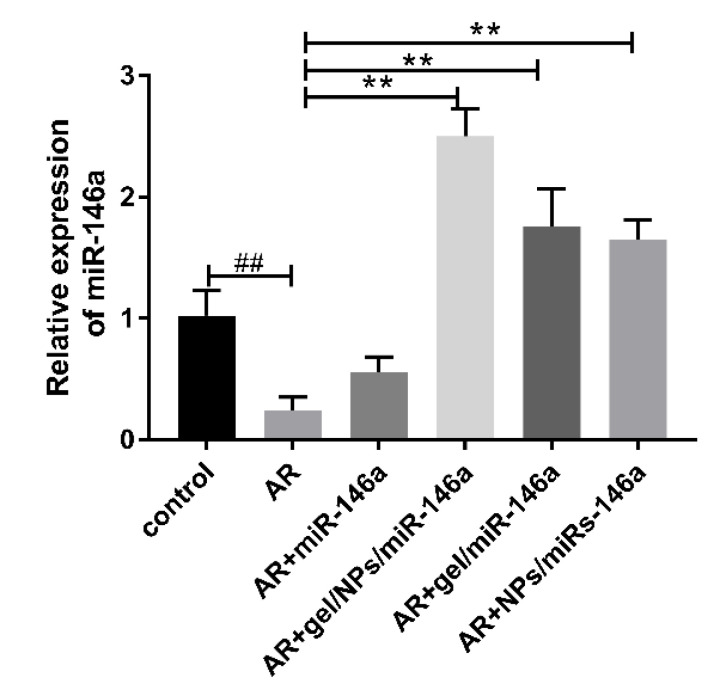
Expression of miR-146a determined by RT-qPCR. Data represent the mean ± SD of three independent experiments. ^##^ *p* < 0.01 compared with the control group; ** *p* < 0.01 compared with the AR group.

Compared with the normal group, the expression of miR-146 in the model group was slightly lower, which was consistent with previous reports (Figure 7). The expression in both the naked miR-146 group and other preparations of miR-146 groups was higher than in the model group, and there was a significant difference between the groups (*p* < 0.05). The expression of miR-146 in the gel/NPs/miR146a group increased significantly. This further confirmed the good effect of the binary formulation on the delivery of nucleic acids. The expression of miR-146 in the nanoparticle group was high compared with the two monophyletic formulations gel/miR-146a and PEG-PLA NPs/miR146a. This showed that the protection of nucleic acids during the delivery of nanoparticles and the release of chemical drugs from the monolithic gel group may be better than the release of nucleic acids (Figure 7).

### 3.9. Western Blot Detection of TLR4 and NF-κB Expression Levels

**Figure 8:**
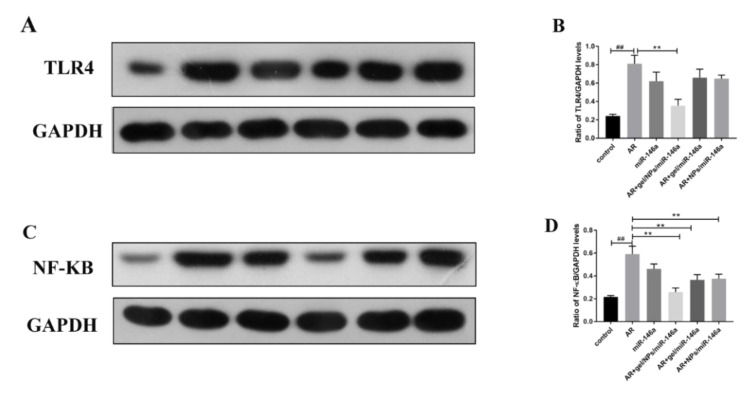
The protein bands of TLR4 (**A**,**B**) and NF-KB (**C**,**D**) normalized to GAPDH in nasal mucosa determined by Western blot analysis. Data represent the mean ± SD of three independent experiments. ^##^ *p* < 0.01 compared with the control group; ** *p* < 0.01 compared with the AR group.

The experimental results showed that compared with the normal control group, the levels of TLR4 and NF-κB in the nasal mucosa of AR rats increased significantly, while both factors were significantly downregulated in AR rats after the delivery of miR-146a by the gel-NPs-miR-146a binary preparation. As expected, downregulation after administration of the gel-NPs-miR146a binary formulation was significantly stronger than the other monophyletic formulations (NPs-miR146a group and gel-miR146a group). Once again, it was verified that the combined effects of the nanoparticles and hydrogel were stronger than any single nucleic acid delivery effect in vivo (Figure 8).

### 3.10. HE Staining

**Figure 9:**
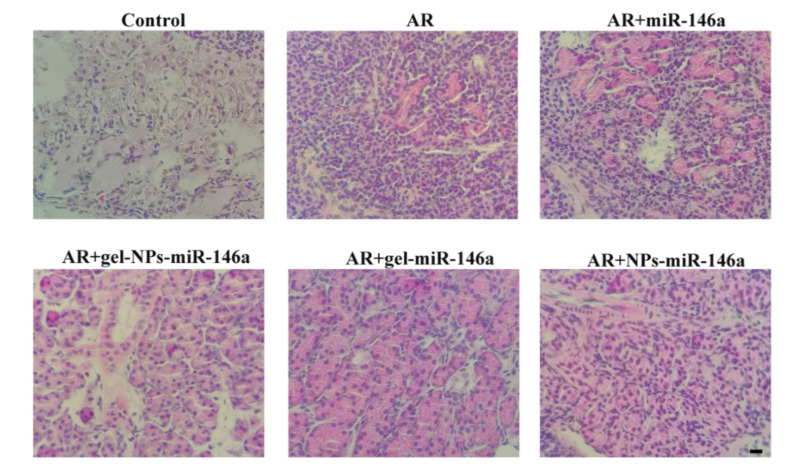
Histopathological changes in the nasal mucosa of rats were investigated by hematoxylin-eosin (HE) staining (400×).

The changes in the histopathological tissue of the nasal mucosa also reflect the success of the modeling (Figure 9). Observation results with 400× light microscopy showed that the overall structure of the nasal mucosa in the normal group was normal and eosinophil and inflammatory cell infiltration were not observed in the lamina propria; however, there was a large number of inflammatory cells infiltration in the model group. The AR + gel-NPs-miR146a rats’ HE results were consistent with histamine release and were also the best performing group among each of the treatment groups. The overall structure of the nasal mucosa in the AR + gel-miR146a group and AR + NPs-miR146a group did not change significantly, but the individual lamina proprias did. There was rare focal inflammatory cell infiltration. Compared with the nude miR-146a group, the binary preparation and the other two monophyletic formulations showed significantly reduced pathological changes in the nasal mucosa in rhinitis model rats. In summary, the pathological results showed that the AR + gel-NPs-miR146a group could significantly reduce the pathological changes i006E the nasal mucosa. Moreover, the AR + NPs-miR146a and AR + gel-miR146a groups had similar pathological effects and improved the nasal mucosa damage caused by allergic rhinitis. The AR + NPs-miR146a group seemed to cause less inflammation, while the AR + gel-miR146a group had improved nasal mucosal cells.

### 3.11. Immunohistochemistry

**Figure 10:**
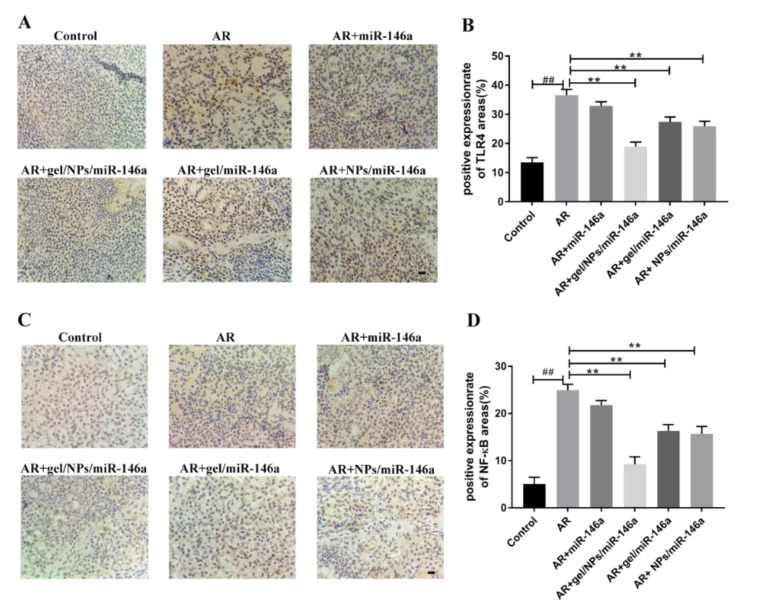
(**A**,**B**) Positive expression of the TLR4 protein in nasal mucosa tissues identified by immunohistochemistry (400×). (**C**,**D**) Positive expression of the NF-κB protein identified by immunohistochemistry in the nasal mucosa (400×). Data represent the mean ± SD of four independent experiments. ^##^ *p* < 0.01 compared with the control group; ** *p* < 0.01 compared with the AR group.

Immunohistochemistry (Figure 10) showed that there was only a small amount of TLR4 and NF-κB expression in the nasal mucosa of the normal group. Compared with the normal group, a large number of brown-yellow particles were observed in the cytoplasm of synovial cells in the nasal mucosa of the model group. Compared with the model group, the expression of TLR4- and NF-κB-positive cells in the nasal mucosa of each dosage from the administration groups decreased, and the decrease in the AR + gel-NPs-miR146a group was the most significant.

## 4. Discussion

The particularity of the intranasal environment determines that intranasal local administration requires a special delivery system. The clearance rate by nasal cilia shortens the retention time of drugs in the nasal cavity and drug absorption is limited. Moreover, naked nucleic acids are blocked from penetrating through the mucosa due to their large negatively charged structure. Therefore, a delivery vehicle is needed to improve the delivery of nucleic acids to antigen-presenting cells [32]. Nanoparticles are currently widely used in drug and gene delivery platforms. Moreover, nanoparticle delivery platforms are increasingly combined with other biological materials to form a hybrid system with novel application prospects [33]. Hydrogels can improve the adhesion of preparations of intranasal drug delivery and have attracted much attention. However, when naked RNA is directly physically dispersed into the gel, most naked RNAs have a burst effect and are quickly released into the surroundings [34]. The stability of miRNA in medium cannot be guaranteed as it is easily degraded by various enzymes in the body. In order to slow down this rapid release and stabilize RNA, after using nanoparticles to wrap the RNA, the nanoparticles can be physically dispersed into a hydrogel to limit their rapid diffusion, thereby delaying the release rate and further improving the stability and effectiveness of the miRNA during delivery [35].

In this study, chitosan was selected as the hydrogel matrix. Chitosan, the product of chitin after removal of the acetyl group, is the main component of the shells, fungi and algae cell membranes of marine arthropods, such as shrimps and crabs, and the shells and bones of mollusks [36]. Chitosan is well known for its outstanding biodegradability, biocompatibility, nontoxicity and physiological functions, such as immune enhancement. Thermosensitive in situ gels have been used to deliver drugs locally into the nose [21]. The degree and speed of the phase change of the in situ gel for nasal administration should not be too slow; otherwise, the semisolid state of the preparation will be too long, and the drug or nanoparticles will rapidly diffuse from the gel and cause a sudden release of the drug. In addition, the nasal gel requires the appropriate strength and mucosal adhesion to allow it to continuously deliver the drug at the administration site, improve the efficiency of nasal absorption and prevent irreversible functional damage to the nasal cilia and mucosa [37]. In order to ensure the adaptability of nasal preparations for nasal administration, the mucosal adhesion degrees of the two prepared gels (chitosan hydrogel and NPs/chitosan hydrogel) were measured, and these preparations were within the desired range. The gel causes little irritation to the nasal cavity and has suitable nasal mucosal adhesion, ensuring that it can deliver nucleic acids stably and continuously. Under physiological conditions, the mucosal adsorption of chitosan polymers has been proven. Because the positively charged amino groups on the chitosan polymer and the anions of the mucus layer (such as sialic acid) produce strong electrostatic interactions, the residence time of the drug in the nasal cavity is prolonged [38]. The results also showed that, although the release time of miR-146 from the gel was prolonged, the use of pure gel to deliver miR-146a produced a retention efficiency in the nasal mucosa that was not ideal. The gel/NPs/miR146a group was more stable than the gel/miR146a and NPs/miR146a groups in vitro, had a higher retention rate in the nasal mucosa and a reduced amount of permeable miRNA. It may be that the NPs/gel retain the nanoparticles in the three-dimensional polymer matrix so that they can be better delivered to the inflamed area and prolong the contact time with the nasal mucosa, leading to a localized effect on the mucosa. The possibility of nanoparticles carrying miR-146a absorbed by a means other than nasal local absorption should be minimal, while exerting a maximum local therapeutic effect in the nasal mucosa. The in vivo results also showed that the AR + gel/NPs/miR146a group had higher expression levels than the AR + gel/miR146a and AR + NPs/miR146a groups and had better pharmacodynamic effects (Figure 8). The nanoparticles protected the nucleic acids to better treat rhinitis.

For the treatment of nasal diseases, it is more desirable for the drug to be absorbed and act on nasal cells while reducing the absorption of the drug into the brain or the whole body. Reducing drug N2B (nasal-to-brain) behavior can be achieved by inducing or increasing efflux transporters, increasing the size of the nanoparticles, adjusting the site of drug deposition in the nasal cavity and reducing nerve cell endocytosis in nasal epithelial cells [39]. In order to achieve better intranasal local delivery, it is necessary to design a reasonable delivery method combined with the treatment purpose. Overall, increasing the size of the nanoparticles is a relatively simple and easy method. In most studies that use PEG-PLA or PEG-PLGA nanoparticles to deliver drugs to the brain, the nanoparticles are often modified with lectins or penetrating peptides to promote absorption. In addition, small particle size (<100~200 nm) is a common feature [40,41]. There are also many studies using chitosan nanoparticles to promote delivery from the nose to the brain, which is associated with the properties of chitosan that can temporarily open the tight junctions of the mucosal epithelium, thereby increasing the penetration of extremely polar compounds (such as peptides, proteins or nucleic acids) [41]. Therefore, we have designed a novel strategy to deliver miRNA by the customized chitosan hydrogel doped with PEG-PLA to better achieve the local treatment and reduce the side effects through other pathways. We chose PEG-PLA nanoparticles with a larger particle size, which can aid in passing through the mucus layer and achieve absorption by the nasal mucosal cells. After administering PEG-modified nanoparticles to deliver nucleic acid for 48 h, both the PEG-PLA NPs and the gel/NPs/miR146a group achieved high Cy3-miRNA-146 retention in the nasal mucosa (Figure 3), but the mucosal permeability of the PEG-PLA NPs group was also high, indicating that there was a risk of transfer to other absorption sites. However, the nanoparticles were uniformly dispersed in the in situ hydrogel to achieve the local, stable and sustained release of miRNA into the nose. The perfect formulation for local administration targeting the nasal mucosa still needs further enhancement. In addition, the reason why miR-146a was selected in this study to be the miRNA that regulates rhinitis symptoms is that miR-146a has no side effects (even in the brain). Unlike miR-155 or miR-466, although miR-146a has the positive effect of interfering with the regulation of rhinitis, it also has an effect on brain nerve cells. Therefore, the treatment of rhinitis requires both binary preparations and selection of appropriate miRNAs.

This study has certain limitations. First, the rat rhinitis model cannot perfectly simulate the symptoms of rhinitis, and the irritation from rhinitis in each rat is not uniform, with individual differences. Additionally, the severity of rhinitis is random. Therefore, the evaluation score and tissue sampling are also random, which interferes with the treatment effects shown in the study. At present, rhinitis is still a niche disease that does not cause substantial harm to the population and has not attracted attention. The application of nanoparticles and water gel in studies of treatments of the disease is low, and although we tried our best, neither good cell simulation nor good simulation of the mucosal system was achieved. It is expected that in future studies, the in vitro cell model and more mature in vitro nasal mucosa model can be used to establish a more perfect in vitro screening method to optimize the preparation parameters.

## Figures and Tables

**Table 1:** Physicochemical characteristics of the nanoparticles.

Formulation	Particle Size (nm)	Zeta-Potential (mV)	PDI	Loading Efficiency (%)
NPs-miR146a	551.5 ± 53.2	−22.6 ± 1.3	0.217	82.8 ± 9.9

**Table 2:** Composition and gelation time at 34 °C of formulations based on mixtures of chitosan with β-glycerophosphate (β-GP).

Formulation Code	CS (*w*/*v*) (%)	GP (*w*/*v*) (%)	Gelation Time (s)
F1	1.5	12	-
F2	1.5	16	-
F3	3	12	330.8 ± 18.4
F4	3	16	290.7 ± 16.2
F5	4.5	12	228.5 ± 13.4
F6	4.5	16	161.7 ± 11.9

**Table 3:** Evaluation parameters for miR-146a in chitosan gel.

Formulation	CS (*w*/*v*) (%)	GP (*w*/*v*) (%)	Gelation Time (s)	Gelation gel Strength (s)	Adhesion Strength (g/cm^2^)	Adhesion Duration (h)
gel/miR-146a	4.5	12	198.5 ± 12.4	42.3 ± 3.6	28.6 ± 2.9	4.9 ± 0.7
